# Transcription Factor Binding Site Positioning in Yeast: Proximal Promoter Motifs Characterize TATA-Less Promoters

**DOI:** 10.1371/journal.pone.0024279

**Published:** 2011-09-09

**Authors:** Ionas Erb, Erik van Nimwegen

**Affiliations:** 1 Bioinformatics and Genomics program, Center for Genomic Regulation and Pompeu Fabra University, Barcelona, Spain; 2 Biozentrum, University of Basel, and Swiss Institute of Bioinformatics, Basel, Switzerland; University of Leuven, Belgium

## Abstract

The availability of sequence specificities for a substantial fraction of yeast's transcription factors and comparative genomic algorithms for binding site prediction has made it possible to comprehensively annotate transcription factor binding sites genome-wide. Here we use such a genome-wide annotation for comprehensively studying promoter architecture in yeast, focusing on the distribution of transcription factor binding sites relative to transcription start sites, and the architecture of TATA and TATA-less promoters. For most transcription factors, binding sites are positioned further upstream and vary over a wider range in TATA promoters than in TATA-less promoters. In contrast, a group of 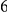 ‘proximal promoter motifs’ (GAT1/GLN3/DAL80, FKH1/2, PBF1/2, RPN4, NDT80, and ROX1) occur preferentially in TATA-less promoters and show a strong preference for binding close to the transcription start site in these promoters. We provide evidence that suggests that pre-initiation complexes are recruited at TATA sites in TATA promoters and at the sites of the other proximal promoter motifs in TATA-less promoters. TATA-less promoters can generally be classified by the proximal promoter motif they contain, with different classes of TATA-less promoters showing different patterns of transcription factor binding site positioning and nucleosome coverage. These observations suggest that different modes of regulation of transcription initiation may be operating in the different promoter classes. In addition we show that, across all promoter classes, there is a close match between nucleosome free regions and regions of highest transcription factor binding site density. This close agreement between transcription factor binding site density and nucleosome depletion suggests a direct and general competition between transcription factors and nucleosomes for binding to promoters.

## Introduction

Large-scale ChIP-chip and protein-microarray experiments, e.g. [1]–[3], have made it possible to identify the sequence specificities of a large number of transcription factors (TFs) in the yeast *Saccharomyces cerevisiae*. The sequence specificities of TFs are generally represented as position specific weight matrices (WMs) and using these WMs in combination with sophisticated comparative genomic algorithms for transcription factor binding site (TFBS) prediction, it is now possible to obtain fairly comprehensive annotations of the TFBSs occurring across yeast promoters [4], [5]. Having such comprehensive TFBS annotations available across promoters genome-wide in turn allows for a rigorous and quantitative study of the ‘grammar’ of this transcriptional regulatory code. Several previous studies have looked at the distributions of the number of binding sites per TF and per intergenic region, co-occurrence of TFBSs for different transcription factors, and similar statistics, e.g. [6]–[9].

Using data from several high-throughput methods, comprehensive annotations of transcription start sites (TSSs) in yeast have also become available recently [10]–[13], and this allows us to study the precise positioning of TFBSs relative to TSSs. In a preliminary study [8], we showed that different TFs show very distinct positional preferences relative to TSS. Here we extend this work by comprehensively studying the positioning of TFBSs relative to TSS across all yeast promoters, and identify novel classes of non-TATA promoters which are characterized by the occurrence of alternative proximal promoter motifs.

TATA sites, also called TATA boxes, which are recognized by TATA-binding protein in mammals and by SPT15 in *Saccharomyces cerevisiae*, are well-known core promoter elements which are known to have very specific positional preferences relative to TSS. In particular, in mammalian promoters the distribution of TATA sites is sharply peaked at about 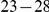 base pairs upstream of TSS [14]. Interestingly, in yeast the distribution of TATA sites peaks much further upstream, i.e. at about 80 base pairs upstream of TSS, and is generally broader [15]. Several lines of recent evidence strongly suggest that, in yeast, the pre-initiation complex (PIC) is recruited further upstream than in mammals, and then ‘scans’ downstream from its place of initial recruitment, until it encounters a initiator site where it then initiates transcription [16], [17]. Although standard textbook descriptions of core promoter architecture often imply that a TATA site is a characteristic feature occurring in promoters in general, recent transcriptome analyses have shown that, both in yeast [15] and in mammals [14], only one sixth to one fifth of all promoters contain a TATA site.

Here we show that, besides the TATA motif, there are 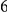 additional ‘proximal promoter motifs’ (PPMs) whose binding sites preferentially occur close to the TSS. By comprehensively comparing the architecture of TATA-containing and TATA-less promoters we show that for most motifs, TFBSs are positioned further upstream and vary over a wider range in TATA promoters than in TATA-less promoters. In contrast, proximal promoter motifs occur preferentially in TATA-less promoters, and their preference for binding proximal to TSS is largely restricted to TATA-less promoters. By studying the sequence preference of the yeast initiator motif we show that all PPMs exhibit sequence similarity to the initiator motif. Moreover, by constructing profiles of the affinity to the initiator motif of sequences upstream of the TSS, we present evidence suggesting that the PIC is initially recruited at the location where TATA is found in TATA promoters, and at the location where other PPMs are found in TATA-less promoters. We show that TATA-less promoters can be classified according to the PPM that they contain, and show that different classes of promoters show different patterns of TFBS positioning and nucleosome coverage. Moreover, we demonstrate that there is a close match between regions of highest predicted TFBS density, and nucleosome free regions, suggesting a general competition between nucleosomes and TFs for binding DNA.

## Results

### TFBS distributions relative to TSS identify seven proximal promoter motifs

As we have described previously [5], [8], [18], we used comprehensive ChIP-chip data in combination with known regulatory sites from the literature and motif finding algorithms, to curate a set of 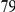 high confidence positional weight matrices representing yeast TFs. As also described previously [19], we have developed sophisticated Bayesian probabilistic algorithms that, given a set of WMs and multiple alignments of intergenic sequences as input, predict TFBSs using explicit models of the evolution of TFBSs, neutrally evolving background sequences, and sequences that are under purifying selection for other reasons. Using these algorithms on multiple alignments of intergenic regions of *Saccharomyces cerevisiae* and the 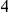 other *sensu stricto Saccharomyces* species that have been sequenced, we predicted TFBSs for the 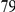 WMs across all yeast intergenic regions.

**Figure 1:**
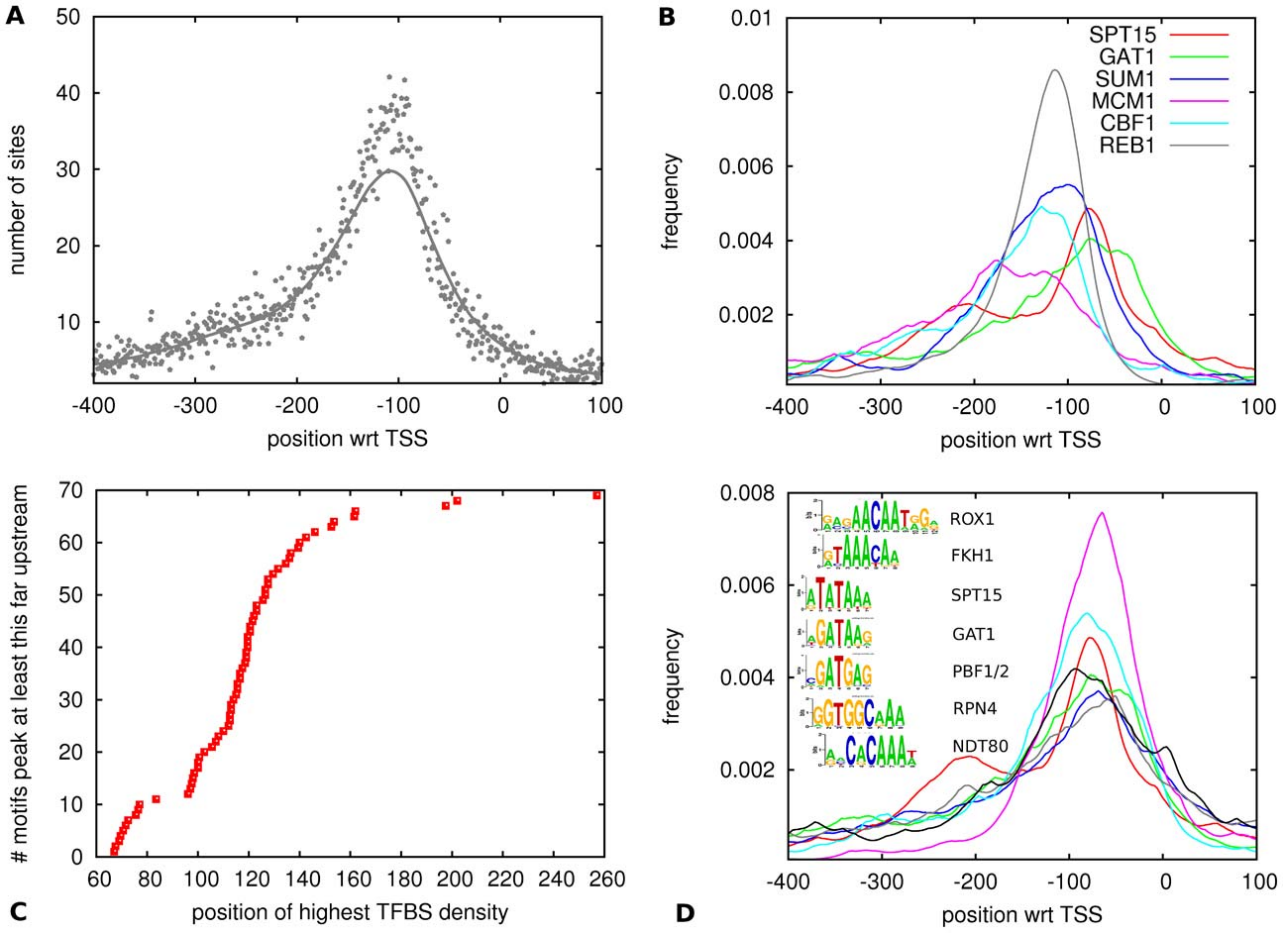
Distribution of predicted TFBSs relative to TSS and proximal promoter motifs. The horizontal axes in panels A, B, and D show the location relative to TSS, where upstream positions are denoted by negative numbers. **A**: The vertical axis shows the total number of sites as a function of position summed over all 79 WMs and all promoter regions, with the solid line showing a smoothed version of the raw distribution (dots). **B**: TFBS distributions for several example TFs. The vertical axis shows smoothed frequency of site occurrence as a function of position for the TFs SPT15 (TATA binding protein), GAT1, SUM1, MCM1, CBF1, and REB1. **C**: Cumulative distribution of the position of highest TFBS density. Each dot corresponds to one motif. Only the 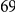 motifs with a sufficient number of predicted TFBSs were used, see Methods. **D**: TFBS distributions relative to TSS for the proximal promoter motifs ROX1 (gray), FKH1 (dark blue), SPT15 (red), GAT1 (green), PBF1/2 (pink), RPN4 (light blue), and NDT80 (black). The inset shows a suggested alignment of the corresponding motifs.

Finally, using experimentally determined TSSs [10]–[12] we then determined, for each TF, the distribution of its binding sites relative to TSS. As shown in Fig. 1A, when summing sites for all TFs, there is a strong peak in TFBS density a little over a 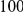 base pairs upstream of TSS, which decays quickly in the first 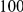 base pairs up- and down-stream of the peak, and shows a more slowly decaying tail further upstream of TSS.

Interestingly, Fig. 1B shows that individual TFs show highly distinct positional profiles with respect to TSS. To investigate this further, we determined for each TF the position at which its binding site density is highest. As shown in Fig. 1C, there is a group of 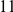 motifs that are neatly separated off from the rest, having a most preferred position between 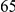 and 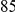 base pairs upstream of TSS. Manual inspection shows that these motifs fall into 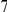 families (Fig. 1D): The well-known TATA motif bound by SPT15, the family of GATA-motifs consisting of GAT1, GLN3, and DAL80, which all bind to a motif containing GATA at its core, the forkhead motif recognized by FKH1 and FKH2, two motifs recognized by PBF1 and PBF2 (previously known as the PAC motif [3], [20]), and the motifs for ROX1, RPN4, and NDT80. In the following we will call these Proximal Promoter Motifs (PPMs). We will refer to the GAT1/GLN3/DAL80 motif as the GATA motif, to the FKH1/FKH2 motif as the FKH motif, and to the PBF1/PBF2 motif as the PBF motif. For the families containing multiple motifs we will restrict our analysis from now on to the motif with the highest number of predicted binding sites genome-wide.

Besides showing the positional distribution of the 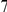 PPMs, Fig. 1D also shows a suggested alignment of the corresponding motifs which we determined by hand. Although the motifs are all different, some clear similarities between the PPMs can also be observed. For example, the cores of the GATA and TATA motifs differ by only 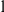 letter, and the PBF motif differs in only 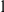 letter from the GATA motif, i.e. GATGAG and GATAAG. The FKH and ROX1 motifs share a common AACAA core, and the CACAA motif of NDT80 differs in only 1 letter from this core. In general, all motifs contain runs of purines interspersed by either a single thymine or a single cytosine. We will see below that the PPMs share these features with the initiator motif found at TSSs.

To investigate whether the positional preferences that we observe could simply be a result of the sequence composition of promoters relative to TSS we performed binding site predictions on a set of randomized alignments. These randomized alignments are constructed by permuting the original alignment columns in such a way as to conserve the di-nucleotide frequencies as a function of position relative to TSS, the exact gap patterns, and the cross-species conservation patterns of the original alignments (see Methods). For example, Fig. S1 shows that the GC-content relative to TSS of the randomized promoters closely matches that of the original alignments. We observe that, across all motifs, the number of predicted binding sites on the randomized alignments is much less than on the true promoters (Fig. S2). This strongly suggests that only a small fraction of our predicted binding sites result from spurious matches to local sequence composition. In addition, we determined the most preferred positions of binding sites on the randomized alignments for each motif and observed that the preferred positions of the PPMs change dramatically, showing that the preferred positions of the PPMs on the true alignments are not a function of local sequence composition (Fig. S3). Finally, as shown in Fig. S4, on the randomized alignments the PPMs show little evidence of preferred positioning relative to TSS at all. Together these results show conclusively that the occurrence of a set of PPMs, and the positional preferences of motifs in general, cannot be explained in terms of di-nucleotide frequencies across promoters.

### Architecture of TATA and TATA-less promoters

**Figure 2:**
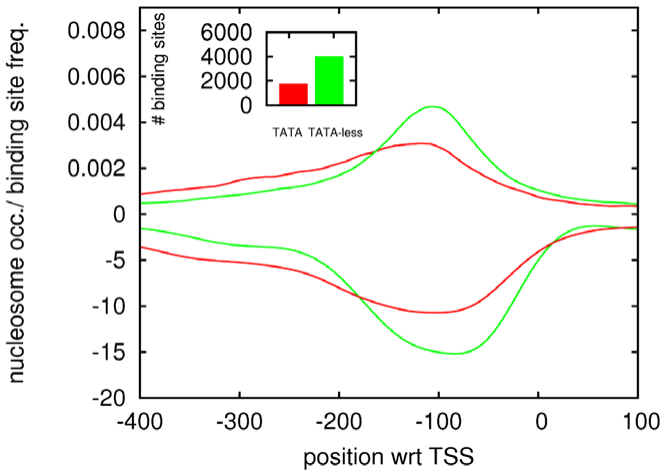
Positional distribution of TFBSs and nucleosomes in TATA and TATA-less promoters. The top two lines show the positional distributions of TFBSs summed over all 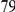 motifs in TATA (red) and TATA-less promoters (green). The inset shows the total number of TFBSs in TATA and TATA-less promoters. The bottom two curves show the average nucleosome coverage (see Methods) in TATA (red) and TATA-less promoters (green).

As we mentioned in the introduction, only between one sixth and one fifth of all yeast promoters contain a TATA site. Given this, we wondered whether the other PPMs may play a role in TATA-less promoters, and whether the TFBS architecture, in general, may differ between TATA and TATA-less promoters. A previous study [15] used a combination of experimental data and computational analysis to determine all TATA promoters in yeast and we used this to evaluate TFBS distributions, separately in TATA and TATA-less promoters. Figure 2 shows that TFBSs occur in general closer to TSS in TATA-less promoters, and that there is a much longer tail of TFBSs occurring further upstream in TATA promoters.

Comparisons of the expression profiles of TATA-containing and TATA-less promoters have indicated that TATA-less promoters are enriched for house-keeping genes that are expressed in a fairly constitutive manner, whereas TATA-containing promoters show more variability in expression, and are often induced in response to various stresses [15], [21]. This ‘inducible’ feature of TATA-containing promoters has been associated with characteristics of their nucleosome occupancy [22], [23], which in general shows more nucleosome coverage immediately upstream of TSS. In parallel to the study of the positioning of TFBSs in different promoters we thus also decided to study nucleosome coverage patterns across different sets of promoters. We produced nucleosome occupancy profiles for each promoter, using data from [24], and Fig. 2 shows the average nucleosome coverage in TATA and TATA-less promoters (see Methods). Our results confirm that TATA promoters have more nucleosome coverage on average. Moreover, the nucleosome coverage closely mirrors the TFBS distributions in that the region of minimal nucleosome coverage is shifted further upstream in TATA promoters, and there is a much longer tail upstream, in contrast to TATA-less promoters where the ‘nucleosome free region’ (NFR) has a more clearly defined position nearer to the TSS.

**Figure 3:**
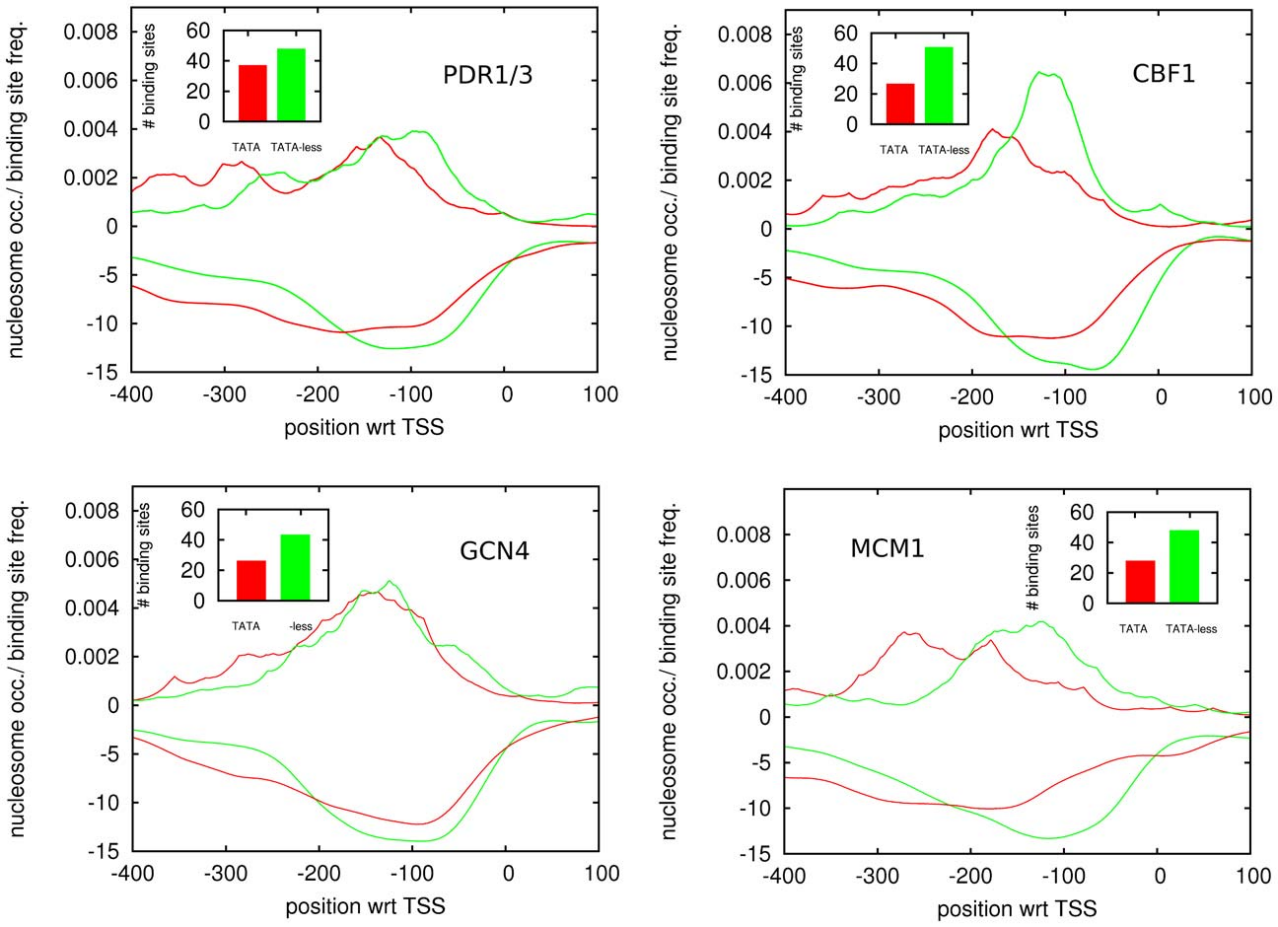
Positional distributions of TFBSs for individual TFs (upper curves) and average nucleosome occupancy profiles (lower curves) for promoters containing at least one TFBS for the corresponding TF, separately for TATA promoters (red) and TATA-less promoters (green). The 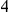 panels show results for the TFs PDR1/3 (top left), CBF1 (top right), GCN4 (bottom left), and MCM1 (bottom right). In each panel, the position relative to TSS is indicated along the horizontal axis and the density of TFBSs (positive values) and nucleosome coverage (negative values) are shown along the vertical axis. The insets show the total number of predicted TFBSs in TATA and TATA-less promoters for the corresponding TF.

We next investigated the distribution of TFBSs for individual TFs in TATA and TATA-less promoters. Consistent with the pattern shown in Fig. 2, for the large majority of TFs we find that the binding sites are positioned further upstream in TATA promoters than in TATA-less promoters. The top two panels of Fig. 3 show two examples of the typical behavior exhibited by most TFs, i.e. the positional distribution of binding sites for PDR1/3 and CBF1 are shifted upstream by a few tens of base pairs in TATA promoters relative to TATA-less promoters.

The bottom two panels of Fig. 3 show examples of the most extreme behavior that we observe. For the TF GCN4 there is essentially no change in the positional distribution of binding sites, whereas for MCM1 the distribution is shifted almost 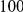 base pairs upstream in TATA promoters.

For each TF we also investigated nucleosome coverage in the subset of promoters that contain a binding site for the TF, again separately for TATA and TATA-less promoters. To this end we averaged nucleosome coverage patterns over all promoters, weighting each promoter with the probability that at least 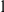 binding site for the TF in question occurs in the promoter (see Methods). Strikingly, we find that, in general, the nucleosome coverage follows precisely the TFBS positioning (Fig. 3). That is, the region of lowest nucleosome coverage generally occurs precisely in the region where the density of TFBSs for the corresponding factor is highest. In particular, the amount of the upstream shift in TATA-containing promoters of the TFBS profiles for the 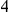 different TFs shown in Fig. 3 is matched closely by the amount by which the region of lowest nucleosome coverage shifts upstream for the subsets of promoters containing binding sites for the corresponding TF. The close match between the nucleosome-free region and TFBS positioning is further supported by Fig. S5, which shows the distribution of TFBSs in subsets of promoters that have their region of lowest nucleosome coverage at different positions relative to TSS.

### Proximal promoter motifs show preferential positioning and binding in TATA-less promoters

The results so far have shown that there is a clear difference in the promoter architecture of TATA-containing and TATA-less promoters. In particular, TFBSs are positioned further upstream in TATA promoters, and the region of lowest nucleosome coverage closely matches the region of highest TFBS occurrence. To investigate the potential role of the PPMs in TATA-containing and TATA-less promoters we measured their binding site positioning in both classes of promoters.

**Figure 4:**
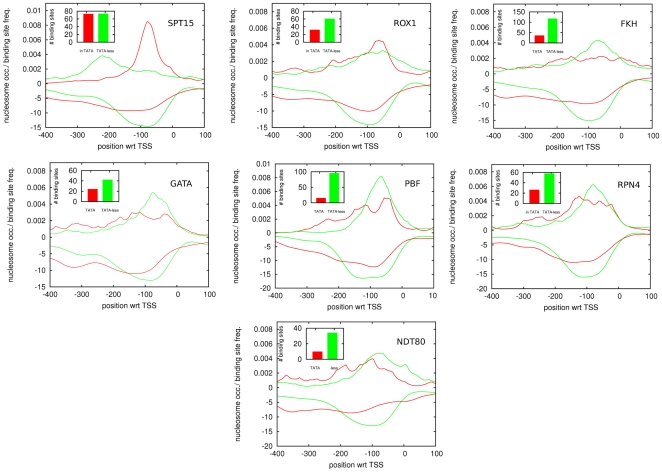
Positional distributions of TFBSs and nucleosome occupancy profiles for proximal promoter motifs in both TATA (red) and TATA-less (green) promoters. Each panel corresponds to one of the proximal promoter motifs. In each panel, the position relative to TSS is indicated along the horizontal axis and the density of TFBSs (positive values) and nucleosome coverage (negative values) are shown along the vertical axis. The insets show the total number of predicted TFBSs in TATA and TATA-less promoters for the corresponding motif.

We first determined binding site positioning for the TATA motif itself in TATA-containing and TATA-less promoters. Figure 4 confirms that SPT15 shows clearest positioning in TATA-containing promoters. Interestingly, in TATA-less promoters a weaker peak in TATA site density is observed further upstream of TSS. Manual inspection has shown that these putative TATA sites occur at the 3′ ends of upstream neighboring genes and overlap TATATA motifs which match so called ‘efficiency elements’ that have been shown to play an important role in polyadenylation in yeast [25]–[27]. It is thus possible that these are false positive predictions resulting from the similarity between TATA sites and polyadenylation efficiency elements. On the other hand, one might speculate that, if the TATA and efficiency element motifs are so similar, SPT15 might in fact bind to these efficiency elements under certain conditions. Indeed recent Chip-chip data [28] confirms that SPT15 binds at the 3′ ends of genes, although this observation has been interpreted to result from circularization of the DNA [29], [30], and the data appear to indicate that the positions of the observed SPT15 binding do not precisely match the location of efficiency elements, i.e. the data suggest strongest SPT15 binding immediately downstream of the 3′ end, whereas the polyadenylation motifs occur immediately upstream of the 3′ end.

We next considered TFBS positioning of the other PPMs. Strikingly, in contrast to other TFs (Fig. 3), the PPMs (with the exception of NDT80) do not show an upstream shift in TATA promoters. Instead, their preferred positioning proximal to the TSS is largely restricted to TATA-less promoters, whereas in TATA promoters there is a much less defined positioning of these PPM sites. The only exception to this trend is ROX1, which shows clearer positioning in TATA promoters. These results strongly suggest that these PPMs play a specific role in TATA-less promoters. Nucleosome occupancy profiles confirm this picture. We classified promoters according to the position of the region with minimal nucleosome occupancy, and calculated TFBSs positional distributions for the PPMs, separately in each of these promoter classes. We find that, with the exception of SPT15, all PPMs have the highest density of TFBSs in the class of promoters that has the region of minimal nucleosome coverage close to the TSS (Fig. S6), which tend to be TATA-less promoters.

As a further support of the preference of PPMs for TATA-less promoters we also investigated directly to what extent the PPMs avoid TATA-containing promoters. For each of the PPMs other than SPT15, and for each TSS, we assigned the PPM to the TSS when a binding site with posterior probability of at least 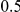 occurred near the preferred TFBS position, i.e. within 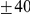 base pairs of the position with highest TFBS density for that PPM. Using a standard hyper-geometric test, we then evaluated whether PPMs are assigned less frequently to TATA promoters than to TATA-less promoters. We find that the FKH, GATA, PBF, and NDT80 PPMs are clearly under-represented in TATA promoters (table 1). For RPN4 and ROX1 we find no significant difference between TATA and TATA-less promoters.

**Table 1:** Under-representation of proximal promoter motif occurrence in TATA promoters.

Motif	p-value
FKH	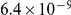
RPN4	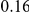
PBF1/2	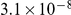
ROX1	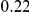
GATA	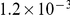
NDT80	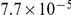

For all PPMS motifs except RPN4 and ROX1, the motifs are statistically significantly more likely to occur in TATA-less than TATA promoters.

### Affinity to the initiator motif in TATA and TATA-less promoters

As mentioned in the introduction, in yeast TATA sites are located significantly further upstream of TSS than in mammalian promoters, and there are various lines of experimental evidence suggesting that the pre-initiation complex (PIC) is initially recruited a substantial distance upstream of TSS, after which it ‘scans’ downstream toward the TSS and initiates transcription at this site, e.g. [16], [17], [31].

**Figure 5:**
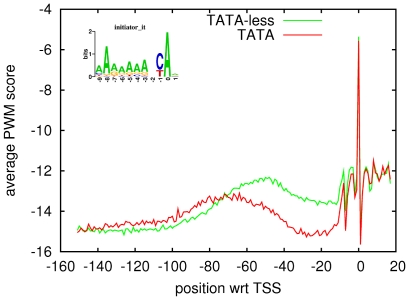
Estimated average affinity profiles for the initiator motif. The horizontal axis shows position relative to TSS and the vertical axis shows the average WM score at the corresponding position, averaged over all TATA (red) and TATA-less (green) promoters. The inset shows the initiator motif. The upstream maximum in TATA promoters corresponds to the preferred position of the TATA motif, and the upstream maximum in the TATA-less promoters corresponds to the preferred position of alternative PPMs in TATA-less promoters.

Besides the TATA motif, another core promoter motif that has attracted considerable attention is the initiator motif, see e.g. [32], [33], which characterizes the sequence patterns at the initiation site. We first checked whether there are any systematic differences between the initiator motif in TATA and TATA-less promoters by constructing initiator motifs separately from TSSs in TATA and TATA-less promoters (see Methods) and found that there is no clear difference between the initiator in TATA-containing and TATA-less promoters. To construct an overall initiator motif for all promoters we proceeded as follows. Using the TSS datasets of [10], [11], we extracted small sequence regions around TSSs that occur in both datasets and which are the unique TSS for their respective promoters. We then build a position-specific weight matrix from these sequences as an initial ‘seed’ for the initiator motif. Next we scored all TSS sequences for this ‘seed’ WM and constructed an updated initiator motif from the 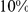 of TSSs with highest score for the seed WM (shown in the inset of Fig. 5). As known from the literature [10], the first base in the transcript (which we denote by zero) is occupied by an A nucleotide, preceded by a C or T. Positions 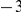 to 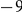 show a clear preference for purines.

The fact that there is no difference between the initiator in TATA-containing and TATA-less promoters suggests that there is no systematic difference in the mechanism of initiation site selection in TATA and TATA-less promoters. Furthermore, we reasoned that just as TF motifs represent the binding specificities of TFs, so the initiator motif may represent the binding specificity of the PIC. Therefore, by systematically comparing the matches to the initiator motif of the sequences upstream of TSS, both in TATA and TATA-less promoters, we may identify which locations upstream of TSS have higher and lower affinity for the PIC, and maybe gain insight into where the PIC is initially recruited.

For every known TSS, from both data sets, we extracted the DNA sequence from 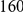 bp upstream to 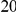 bp downstream (whenever the intergenic region was long enough) and recorded the WM score to the initiator motif at each position. We show the average of several thousand profiles obtained this way in Fig. 5, separately for TATA and TATA-less genes. We imagine that the profiles in Fig. 5 indicate the average sequence affinities (or binding energies) of the PIC to the sequences at different positions, so that the PIC will spend more time in areas where the affinity is high, and scans (or diffuses) more quickly through areas where the affinity is low. As expected, we find a strong peak in the PIC affinity at the TSS in both promoter classes. Interestingly, both promoter classes then show a minimum in PIC affinity in the region immediately upstream of the TSS, and a second maximum in PIC affinity further upstream.

Importantly, the locations of these second maxima clearly distinguish TATA-containing promoters from TATA-less promoters, with the maximum occurring significantly further upstream in TATA promoters. The affinity profiles are suggestive of a process in which the PIC is initially recruited at the upstream peak, after which it would quickly scan through the low affinity region, to finally ‘lock in’ at the strong initiator motif at the TSS. Most importantly, however, from the point of view of PPM analysis, is the fact that the location of the upstream peak in PIC affinity in the TATA promoters corresponds to the position where the highest density of TATA sites is found (see Fig. S7), and the location of the upstream peak in PIC affinity in TATA-less promoters corresponds to the location where the highest density of the other PPMs is found. This result suggests that these other PPMs may interact directly with the PIC in TATA-less promoters.

Finally, we note that, although not very specific, there is a clear similarity between the initiator motif and the PPMs: all of these motifs contain runs of purines interrupted by a pyrimidine (see Fig. 1D). This is consistent with the hypothesis that the PIC is initially recruited to the area where PPMs are found in both TATA and TATA-less promoters.

### Classifying promoters by PPM

As a final comparison of the PPMs we classified promoters according to the PPMs that they contain. For each TSS we calculated a score for each PPM based on the quality and the position of sites for that PPM (see Methods). We then assigned each TSS to the PPM with the highest score. Note that a TSS can remain without an assigned PPM if no PPM motif occurs in an appropriate position upstream of the TSS. To test the robustness of our results to changes in TFBS prediction methodology, we obtained results both using a ‘lenient’ setting of parameters that maximizes sensitivity and a ‘strict’ parameter setting that maximizes specificity (see Methods). As shown in table 2, whereas the number of promoters that are not assigned to any PPM depends strongly on whether sensitive or specific TFBS predictions are used, the relative fractions of TSSs assigned to different PPMs vary much less. In particular, about 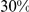 of TSSs that have an assigned PPM are assigned to the TATA motif, and the TSSs are relatively evenly divided among the other PPMs.

**Table 2:** Classification of TSS.

Motif	specific	sensitive
no PPM	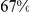	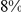
TATA	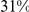	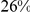
FKH1	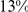	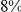
RPN4	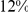	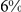
PBF1/2	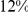	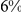
ROX1	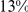	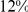
GAT1	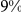	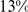
NDT80	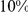	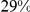
Total non-TATA	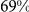	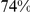

Fractions of all TSSs assigned to different proximal promoter motifs using parameters that produce either specific or sensitive predictions. The percentage of ‘no PPM’ in the first row is with respect to the set of all TSS. All other percentages are with respect to the subset of TSSs that have a PPM assigned.

**Figure 6:**
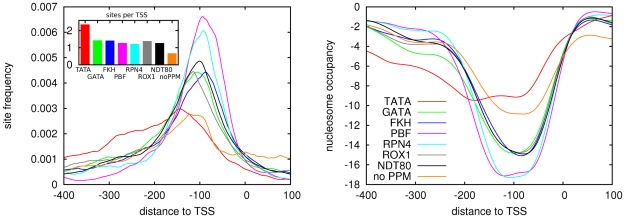
Positional distribution of TFBSs and nucleosomes in promoters assigned to different proximal promoter motifs. Left panel: Overall positional distributions of TFBSs (summed over all 79 motifs) relative to TSS in the sets of promoters assigned to different PPMs. The inset shows the average number of TFBSs per promoter for each of the PPMs. Right panel: Average nucleosome coverage profiles for the sets of promoters assigned to different PPMs.

We then determined the overall frequency, positional distribution of TFBSs, and nucleosome coverage, for each set of TSSs assigned to one of the PPMs, and for the set of TSSs that are not assigned to any of the PPMs. Strikingly, the profiles that we obtain clearly identify three classes of promoters (Fig. 6). Promoters assigned to PBF or RPN4 show the narrowest distribution of TFBSs and these promoters also show by far the strongest nucleosome free region. Promoters assigned to GATA, FKH, ROX1, and NDT80 form the second class with peaks in TFBS density that are less steep, and nucleosome free regions that are correspondingly less deep than those for PBF and RPN4 promoters. Finally, promoters assigned to TATA and those having no PPM assigned show the weakest positioning of TFBSs and show by far most nucleosome coverage. It is also interesting to note that, among all classes of promoters, TATA promoters have the highest overall frequency of TFBSs, whereas promoters without any PPM assigned have the lowest overall frequency of TFBSs. In summary, by classifying promoters according to the PPM they contain, we find that different classes of promoters show clearly distinct TFBS positioning and corresponding nucleosome coverage profiles.

## Discussion

Starting from a genome-wide annotation of TFBSs for 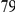 yeast regulatory motifs, we comprehensively studied TFBS positioning and nucleosome coverage profiles across *Saccharomyces cerevisiae* promoters. We uncovered that TATA-box containing and TATA-less promoters have significantly different architectures. Compared to TATA-less promoters TATA promoters show an overall lower number of TFBSs per promoter, these TFBSs occur further upstream of TSS on average, and show a wider distribution of distances with respect to TSS. We find that the TFBS profiles closely mirror nucleosome coverage profiles, i.e. TATA promoters have higher nucleosome coverage, the region of lowest nucleosome coverage occurs further upstream, and the region of lowest nucleosome coverage is more sharply defined in TATA-less promoters.

There recently has been a large amount of investigation into the mechanisms that determine nucleosome positioning, and the extent to which nucleosome positioning is determined by intrinsic sequence preferences of the nucleosomes is currently actively disputed, see [34]–[38]. Since nucleosome positioning is not the main topic of this work, we do not wish to enter into this debate here. However, we do note that the remarkably close and consistent match that we observed between TFBS density profiles and nucleosome coverage profiles across different subsets of promoters, strongly suggests that competition between TFs and nucleosomes for binding to DNA likely plays an substantial role in shaping nucleosome occupancy profiles in yeast promoters.

Whereas the position of overall highest TFBS density occurs more than 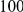 bps upstream of TSS, the TATA motif itself has highest density more proximal to TSS, i.e. at approximately 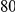 bps upstream of the TSS. This is still considerably further upstream than the location of the TATA-box in mammals, where it occurs about 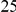 bp upstream of TSS, and there is considerable evidence [16], [17] that, in yeast, the PIC is recruited significantly upstream of TSS and then ‘scans’ down the upstream sequence until it encounters the site where it initiates transcription. To investigate this scanning process we constructed an initiator motif, i.e. representing the sequences at the initiation site, and established that it is essentially identical in TATA and TATA-less promoters. Moreover, in TATA promoters the initiator motif has a maximum both at the TSS and at the position of highest density of TATA sites, suggesting that the PIC may initially be recruited to the position of the TATA sites, and start its scanning from this position. We also saw that in TATA-less promoters this peak in affinity of the initiator motif occurs closer to the TSS.

**Figure 7:**
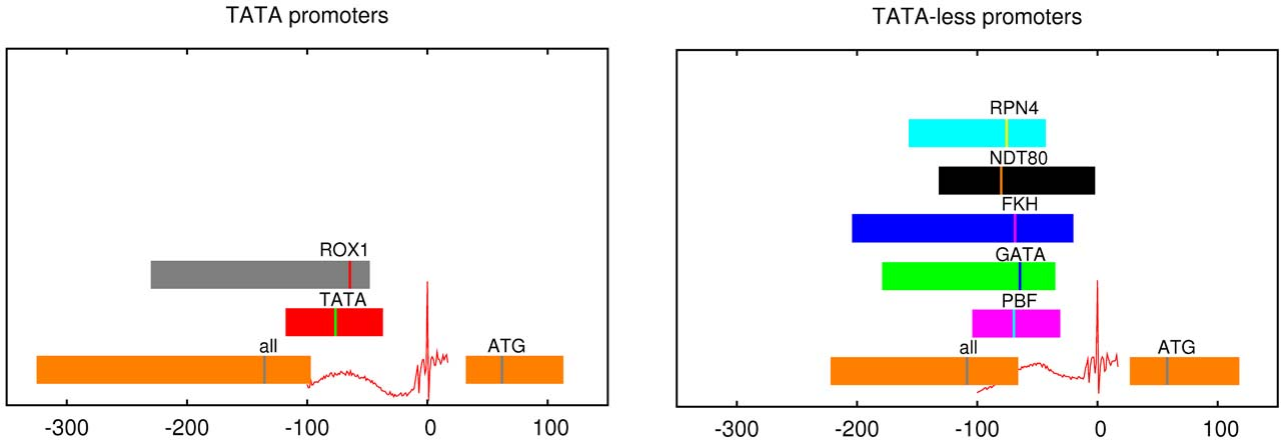
Diagrammatic summary of the architecture of TATA (left panel) and TATA-less (right panel) promoters. Indicated are the maximum and inter-quartile range of the distributions of all TFBSs and the TFBSs for individual PPM motifs. Also shown are the affinity profiles with respect to TSS of the initiator motif, and the distribution of the position of the translation start codon.

A key result of this study is that, besides the TATA motif, there are an additional 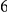 regulatory motifs that also preferentially occur proximal to TSS, i.e. between 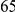 and 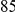 bps upstream of TSS. These alternative proximal promoter motifs occur preferentially in TATA-less promoters and their positioning proximal to TSS is observed predominantly in TATA-less promoters. Moreover, the position of highest density of alternative PPMs in TATA-less promoters corresponds to the position at which the second maximum in initiator affinity occurs, suggesting that, just as the PIC is initially recruited to the TATA site in TATA promoters, in TATA-less promoters the PIC may be initially recruited to alternative PPMs. In addition, we showed that TATA-less promoters can be classified based on the PPM they contain, and that different classes of TATA-less promoters show distinct TFBS and nucleosome coverage distributions. Figure 7 provides a diagrammatic summary of the differences in architecture of TATA and TATA-less promoters identified in this study.

Our results establish that many of the TATA-less promoters are characterized by the occurrence of alternative PPMs, and suggest that these play a crucial role in regulating transcription at these TATA-less promoters. The main question that now arises is what the precise functional role of these alternative PPMs is, and how their function relates to that of the TATA-box. With respect to the latter, although only about 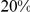 of promoters contain a TATA-box, the TATA binding protein SPT15 is recruited to all promoters, and is generally required for transcription [39], [40]. Although the precise mechanism of function of the TATA site remains elusive, it is clear that at TATA promoters the TATA site is required for proper transcription [15], and one could imagine that the TATA site is requirement for recruitment of the PIC.

The simplest hypothesis for the functioning of the alternative PPMs, which is consistent with all our results, is to assume that they ‘replace’ the TATA site in TATA-less promoters, i.e. that these PPM sites are directly involved in recruiting the PIC. However, a review of the literature on the PPM motifs is at odds with this simple interpretation.

First, GATA sites are generally found upstream of genes that are subject to nitrogen catabolite repression [41]. Very roughly, in nitrogen-rich media these sites are bound by the repressors DAL80/GZF3 while in nitrogen-poor media the sites are bound by GAT1 and GLN3, activating their target genes. Moreover, the signaling of nitrogen availability is mediated by the TOR1 complex [42] with both GLN3 and GZF3 interacting directly with Tor1p [43], [44]. In addition, there is a significant amount of cross-regulation between the GATA factors themselves, including binding to each other's promoters [44]. Thus, activator and repressor GATA TFs compete for binding to the GATA sites, and depending on nitrogen availability this competition will favor either the activating or repressing factors. Thus, although there is a reported case in the literature of a GATA site being recognized by TATA binding protein [45], the main function of the motif appears to be in mediating either repression of activation of genes in response to nitrogen levels.

The NDT80 motif is another example of a motif where a repressor and an activator TF compete for binding to target sites. NDT80 is a meiosis-specific TF that is required for exit from pachytene and that activates middle sporulation genes. During mitosis and in the vegetative state the same binding sites, which are also called middle sporulation elements (MSEs), are bound by the repressor SUM1, i.e. SUM1 acts as a brake on meiosis [46], [47]. Thus, as for the GATA motif, in nutrient-rich conditions the target sites are bound by a repressor, whereas under starvation, when the cells go into sporulation, the repressor is replaced by an activating TF.

A similar function applies to the third proximal promoter motif, ROX1. ROX1 is a heme-dependent repressor of hypoxic genes, i.e. under aerobic conditions ROX1 binds to its target sites while under anaerobic conditions its targets are derepressed [48]. However, the fact that ROX1 is associated mostly with TATA promoters makes it a somewhat special case.

Fourth, the forkhead transcription factors FKH1 and FKH2 are key regulators of the cell-cycle in yeast, targeting the CLB2 cluster of genes which includes the downstream TFs SWI5 and ACE2 [49]. The two forkhead factors often compete for binding to the same promoters [50] and interact with different chromatin remodeling complexes to repress target genes during the G2/M and G1 phases of the cell cycle [51]. Thus, like in the previous examples, the forkhead TFs can act as repressors on their targets, effectively implementing a check-point that is released when they are displaced from their target sites.

Fifth, the PAC (Polymerase A and C) motif is found in the promoters of ribosome biogenesis and rRNA genes and it has recently been shown to be bound by the TFs PBF1 and PBF2 [3], [20]. It has become clear that both PBF1 and PBF2 act as repressors on their targets genes and are activated upon stresses such as heat shock or nutrient signals, with the two TFs being responsive to different stress signals [52]. It is as of yet not clear whether any other (activating) TF may bind to PAC sites under nutrient-rich conditions. The PBF motif thus seems to implement a similar check-point on nutrient availability, releasing its target ribosome biogenesis genes from repression when sufficient nutrients are available.

Finally, RPN4 is an activator of 26S proteasome genes which is itself rapidly degraded by the proteasome, generating a negative feed-back loop that controls proteasome homeostasis [53]. RPN4 expression is controlled by stress responses and the feed-back loop between RPN4 expression and the proteasome is important for cell viability under various stresses [54]. Thus in contrast to all other examples which involved binding by either repressors or competition between repressing and activating TFs for binding to the PPM, the RPN4 sites seem to be mainly targeted by the activator RPN4. However, RPN4 clearly plays a role in response to various stresses.

In summary, it appears that all PPMs are involved in responding to environmental stresses, often involving nutrient availability, either releasing (GATA, NDT80, ROX1) their target genes in response to the stress or (PBF, FKH) repressing their targets when stresses are present. Another feature shared by the PPMs is that, through competition of both activating and repressing TFs binding to the site, the PPM sites are essentially always bound. These features are consistent with their preferred targeting of TATA-less promoters.

Previous studies have shown that TATA promoters in yeast are characterized by closed chromatin, regulation through chromatin, and that many of the associated genes are upregulated upon various stresses. In contrast, ‘house-keeping’ genes tend to have TATA-less promoters [15]. Simplifying one might say that, under nutrient-rich conditions, TATA promoters are ‘off’ by default and the TATA boxes are occluded by nucleosomes. In contrast, many of the TATA-less promoters are expressed and have a distinct nucleosome free region proximal to the TSS [23]. Upon the appearance of various stresses many of the TATA promoters are induced whereas many of the TATA-less promoters are repressed. The alternative PPMs identified in this study appear to generally be involved in this switching in response to nutrient availability and other stresses. Our results suggest that, whereas TATA promoters may respond to a large diversity of stresses, the alternative PPMs may be involved with responding to specific stresses such as cell-cycle check points (FKH), nitrogen and carbon levels (GATA, NDT80, PBF), oxygen levels (ROX1), and heat shock (PBF). Whereas TATA sites may be occluded by nucleosomes in nutrient-rich conditions, most of the PPM sites switch between accommodating repressing and activating TFs, and are thus generally associated with regions depleted of nucleosomes.

## Materials and Methods

### Binding site annotation

The binding site annotations were performed as described previously [8]. Briefly, for each intergenic region in *Saccharomyces cerevisiae* orthologous intergenic regions from four other *sensu stricto Saccharomyces* species were obtained using the ORF annotations of [55], [56]. We used T-Coffee [57] to multiply align the intergenic regions. We obtained the set of weight matrices used in this study by running PhyloGibbs on intergenic alignments of regions bound by a common transcription factor using ChIP-chip binding data [2] as described in [18]. For constructing the PAC motif [58] bound by PBF1 and PBF2, PhyloGibbs was run on ribosomal genes. Finally, additional WMs were obtained by curating the SCPD collection of experimentally determined binding sites [59] using the PROCSE algorithm [60]. We then annotated binding sites for all WMs by running the MotEvo algorithm [19] on the multiple alignments of all intergenic regions.

The randomized alignments were constructed as follows. Since we want to maintain the exact conservation patterns of the original alignments we first checked which subset of species have orthologs for each of the alignments and find that for 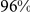 of our regions, 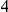 or all 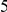 of the species were present (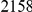 intergenic regions in all). The other 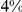 of alignments were discarded. We went through all alignment columns of all original alignments and divided them according to the subset of species present in the alignment, the gap pattern of the alignment column, the position relative to TSS of the column, and the nucleotide that occurs immediately upstream. We then went through the original alignments again and replaced each alignment column with a randomly sampled column from the same position relative to TSS, having the same subset of species, the same gap pattern, and the same neighboring nucleotide as the original column. In this way randomized alignments were created with the same position-dependent nucleotide frequencies, the same species present, the exact same gap patterns, and the exact same conservation statistics.

### Occupancy profiles

We used a combination of the TSS annotations from [10]–[12] to build distance distributions of TFBSs and nucleosome occupancy relative to TSS. To avoid ambiguity, we used only those intergenic regions that are upstream of a single gene to construct the positional distributions of TFBS and nucleosomes, i.e. divergently transcribed intergenic regions were discarded. Usually multiple TSSs were given per region, in which case we assigned a probability to each TSS according to its abundance, separately normalizing the TSSs of different data sets. For the TFBS distributions, we determined the distance 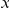 from the center of each TFBS to the TSS and added the product of the posterior probability of the TFBS and the probability of the TSS to the total number of sites 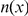. For nucleosome occupancy profiles, we used 4 bp resolution tiling-array data from [24]. Nucleosome occupancy profiles for individual motifs were determined by multiplying the nucleosome occupancy at a given position relative to TSS not only by the probability of the TSS but also by the probability that the region contains a site for the motif. All distributions were smoothed using a double-exponential kernel of width 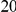 bp and normalized over a range from 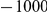 bp to 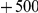 bp relative to TSS. The values for nucleosome occupancy shown in the figures are log ratios of tiling-array hybridizations per considered promoter smoothed with the kernel function.

To determine the most preferred position of a motif in a way that is robust to the noise in the positional profiles we proceeded as follows: We first determined the position 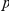 with global highest density and then found the positions 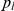 and 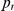 to the left and right of 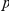 where the TFBS density had fallen to 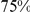 of the maximum value. The interval from 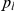 to 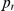 roughly corresponds to the center of the ‘peak’ in TFBS density and we chose the middle of this region, i.e. 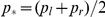 as the most preferred position for the motif. For motifs with too few annotated TFBSs the positional profiles are too noisy to reliably determine a preferred position. Therefore, we discarded motifs for which the total number of annotated TFBSs, which is given by the sum of the posterior probability of all sites, was less than 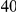. After this filtering 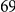 of 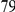 motifs remained. For the test with randomized columns the same 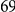 motifs were used (independent of the number of predicted sites for these motifs).

### Initiator affinity

To start constructing initiator motifs we wanted to initially focus on genes that have the same *unique* TSS in all data-sets used [10], [11]. However, there are only 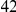 such unique consistent TSSs, illustrating that TSS usage is typically varied. Moreover, 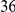 of the 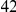 unique consistent TSSs occur in TATA-less promoters (Figure S8) which leaves too few TSSs in TATA promoters to construct even an initial initiator motif for TATA promoters. For TATA promoters we thus relaxed the conditions to obtain more sequences: The TSS had to be unique for the gene in one data set and contain more than 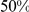 of the probability in the other set. This way the TATA set yielded 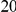 sequences (Fig. S8). We iteratively updated the TATA and TATA-less initiator motifs by collecting all TSSs of the combined data sets (separately for TATA and TATA-less genes) for which the initiator WM scored better than background. Sites for the final initiator motifs were found for 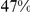 of TATA TSS and 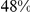 of TATA-less TSSs (Fig. S8).

Finally, after we determined that there were no systematic differences between the initiator motifs in TATA and TATA-less promoters (relative entropies between the two WMs are around 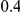 bits, which is less than 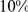 of their average information content), we also constructed a single overall initiator motif by starting from the 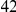 initial consistent TSSs, and collecting the 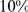 of TSSs genome-wide that had highest WM score for this motif. The roughly 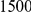 resulting sequences were used to obtain the final initiator WM (inset in Fig. 5).

To obtain the affinity profiles, for each TSS separately and in all regions, the sequence from −160 bp to +20 bp relative to the TSS were extracted (if the intergenic region was long enough). This gave 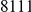 sequences for TATA-less promoters and 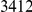 for TATA promoters. Then, at each position relative to TSS, we averaged the WM score (i.e. the log-probability of the sequence given the WM) over all sequences.

### TSS assignments

For the assignment of PPMs to TSSs we consider only TFBSs, for each PPM, that occur near the preferred position for the PPM. For each PPM, we considered TFBSs within a range of 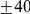 bps from the most preferred position for the PPM. In addition, for each PPM we determined a positional profile over this 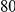 bp range, i.e. the relative probabilities of TFBS occurrence for the PPM within this 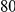 bp range. For each TSS we calculated a score for each PPM by summing over all TFBSs for the PPM, the product of the posterior probability of the site and the positional probability of the site. The TSS was then assigned to the PPM with the highest score. TSSs that remain without an assigned PPM are thus those for which no site for any of the PPMs occurs in the 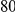 bp ranges of each PPM.

In addition to the standard MotEvo settings which yielded the ‘specific set’ of TFBS predictions, we also produced a set of more ‘sensitive’ TFBS predictions. In making its predictions, MotEvo considers that each region within the multiple alignment can either contain a binding site for one of the WMs (which has been under selection in one or more of the species), neutrally evolving background DNA, or a regulatory element of unknown function, i.e. a TFBS for a TF for which we currently have no WM [19]. This avoids that MotEvo makes false positive predictions on regions that show only weak similarity to the WM but that are very well conserved. In the sensitive setting the prior probability assigned to such ‘unknown motifs’ is reduced while the prior probabilities for the PPMs are increased.

## Supporting Information

Figure S1 **Average GC-content of the true (red) and randomized (green) promoter sequences as a function of position relative to TSS.** The figure shows that, in line with the way the randomized promoters were constructed (see Methods), the GC-content of the randomized promoters closely tracks that of the original promoters.
(TIFF)

Figure S2
**Reverse-cumulative distribution of the number of predicted binding sites across motifs for predictions on the original alignments (red symbols) and on the randomized alignments (green symbols).** The ‘number’ of predicted binding sites is defined as the sum of the posterior probabilities of all binding sites that lie within the 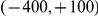 region relative to TSS on the 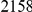 alignments that were used for creating the randomized set. We observe much smaller numbers of binding sites on the randomized promoters, e.g. only about 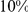 of the motifs on the randomized alignments have more predicted sites than the motif with the *least* predicted sites on the true alignments.(TIFF)

Figure S3 **Cumulative distribution of the position of highest TFBS density across motifs for binding site predictions done on the true (red) and randomized (green) alignments.** Each symbol corresponds to one motif. The blue symbols indicate the PPMs on the randomized alignments. As the figure shows, on the randomized alignments the positions of highest TFBS density for the PPMs vary greatly indicating that their preference for proximal locations in the true alignments is not a consequence of di-nucleotide composition of the promoters.
(TIFF)

Figure S4 **Positional distributions of TFBSs for the proximal promoter motifs on the true (red lines) and randomized (green lines) alignments.** Each panel corresponds to one of the proximal promoter motifs. In each panel, the position relative to TSS is indicated along the horizontal axis and the density of TFBSs is shown along the vertical axis. The figures show that PPMs show little evidence of preferred positioning on the randomized alignments.
(TIFF)

Figure S5 **Positional distributions of all TFBSs for promoters with the region of minimal nucleosome coverage (MNO) up to 100 base pairs upstream of TSS (red), between 100 and 200 base pairs upstream (green), between 200 and 300 base pairs upstream (blue), and more than 300 base pairs upstream (purple).** The inset shows the total number of binding sites in each of the promoter classes. The results demonstrate the locations of highest TFBS density match the locations of the region of minimal nucleosome occupancy.
(TIFF)

Figure S6 **Positional distributions of the TFBSs for each proximal promoter motif, separately in promoters with the region of minimal nucleosome occupancy (MNO) up to 100 base pairs upstream of TSS (red), between 100 and 200 base pairs upstream (green), between 200 and 300 base pairs upstream (blue), and more than 300 base pairs upstream (purple).** Each panel corresponds to one proximal promoter motif (first row: TATA, ROX1. Second row: FKH, GATA. Third row: PBF, RPN4. Last row: NDT80). In each panel the horizontal axis shows position relative to TSS and the vertical axis shows TFBS density. The insets show the total numbers of predicted TFBSs in each promoter class for the corresponding motif. With the exception of the TATA motif, which shows highest density of TFBSs in promoters with MNOs more than 200 base pairs upstream, all other PPMs show highest densities of TFBSs in promoters with MNOs more proximal to TSS.
(TIFF)

Figure S7
**Affinity profiles for the initiator WM exclusively for TATA promoters.** To construct the red profile, the TSS was taken as reference point in each promoter, whereas for the green profile the TATA box was taken as a reference point. To align the green and red profiles, we set the TATA-box reference point at the position where the highest density of TATA sites is observed (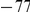 bp relative to TSS). The results demonstrate that the second maximum of the red profile, at around 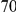 bps upstream of TSS, corresponds to the affinity of the initiator motif for a region around that TATA-box.(TIFF)

Figure S8
**Upper panels: Seed WMs for initiator motifs in TATA and non-TATA promoters.** Lower panels: Iterated WMs obtained from 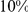 of the TSS sequences scoring best using the WMs in the upper panels.(TIFF)
