# Supplementary figures and images for: Transcription Factor Binding Site Positioning in Yeast: Proximal Promoter Motifs Characterize TATA-Less Promoters
Source: PLoS One. 2011 Sep 9;6(9):e24279. doi: 10.1371/journal.pone.0024279 (PMC3170328; doi:10.1371/journal.pone.0024279)

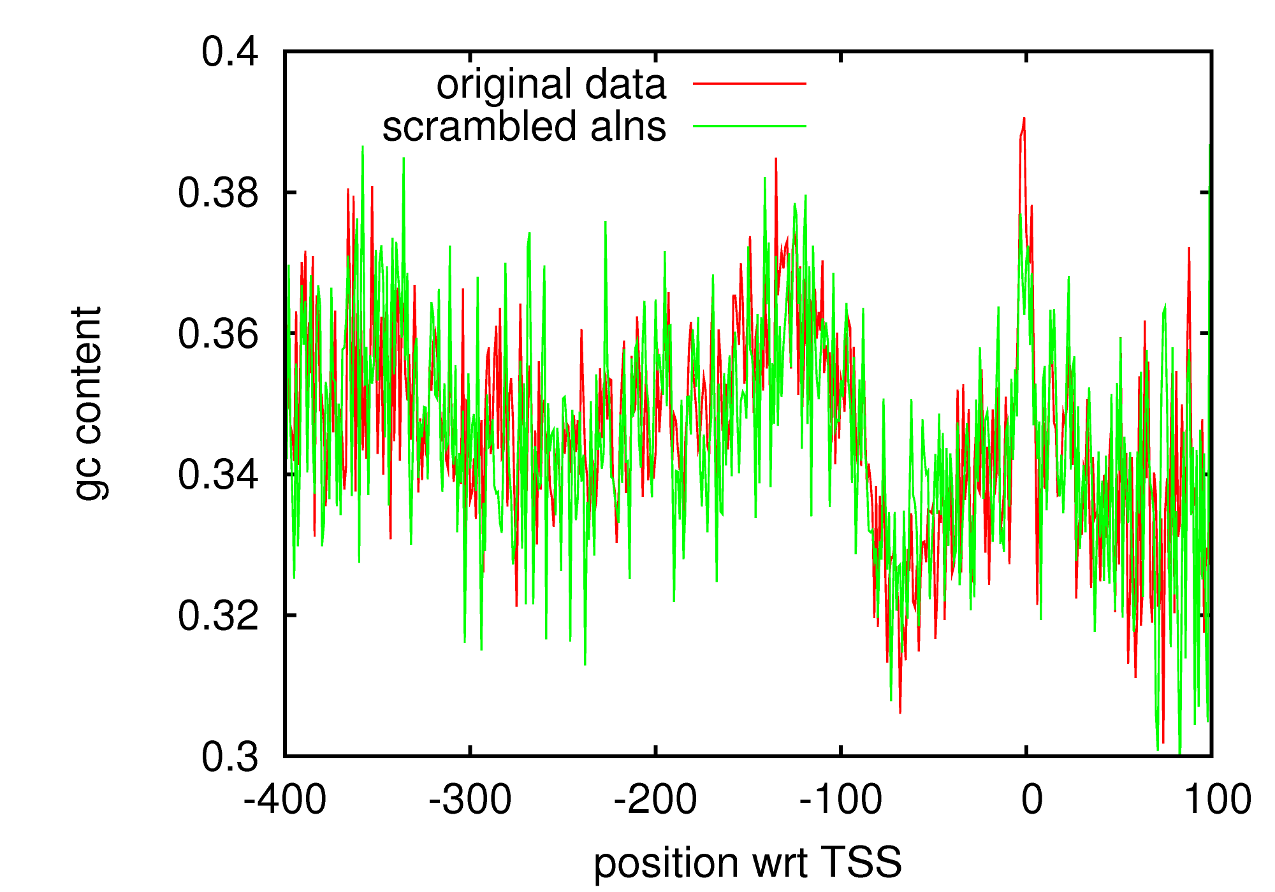

Supplement: Figure S1 — Average GC-content of the true (red) and randomized (green) promoter sequences as a function of position relative to TSS. The figure shows that, in line with the way the randomized promoters were constructed (see Methods), the GC-content of the randomized promoters closely tracks that of the original promoters. (TIFF) [file pone.0024279.s001.tiff]

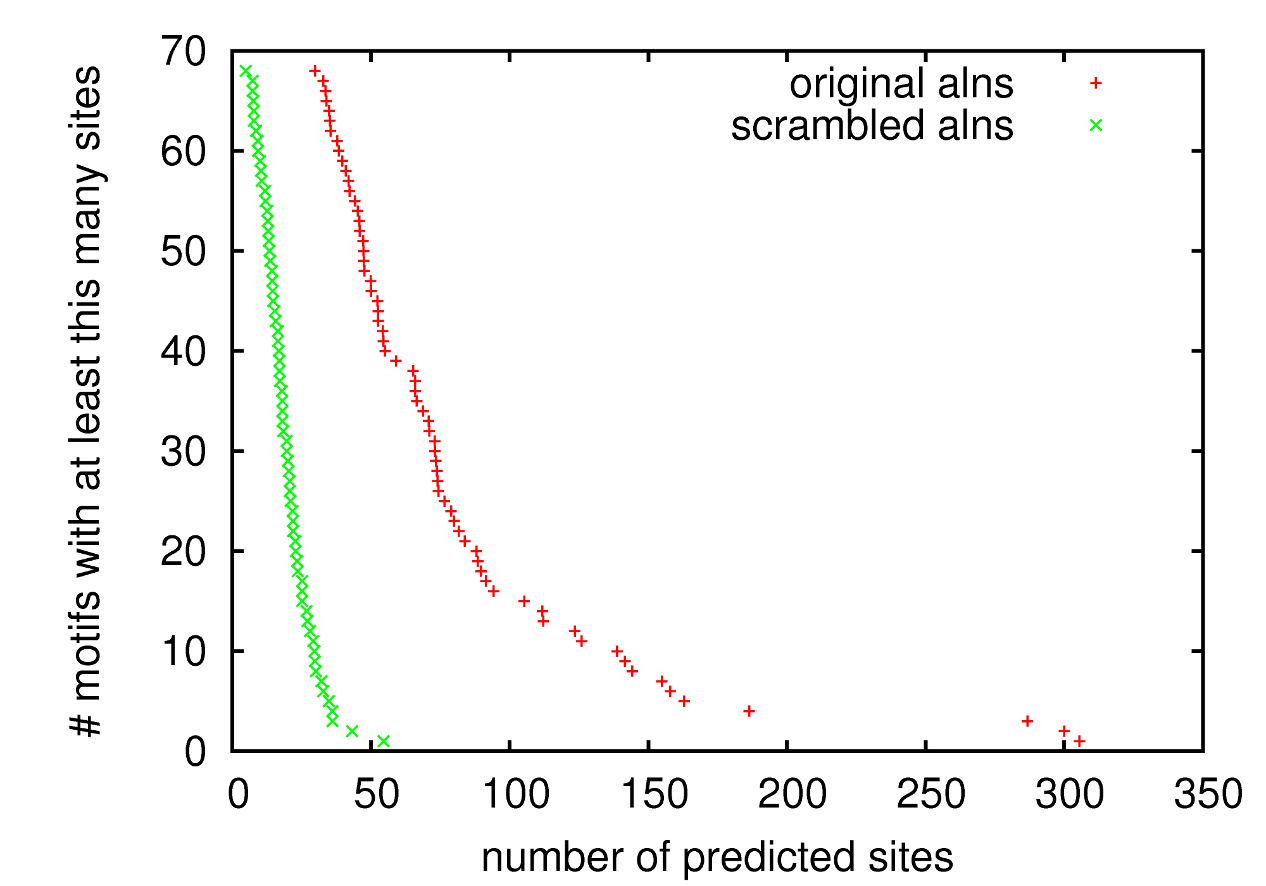

Supplement: Figure S2 — Reverse-cumulative distribution of the number of predicted binding sites across motifs for predictions on the original alignments (red symbols) and on the randomized alignments (green symbols). The ‘number’ of predicted binding sites is defined as the sum of the posterior probabilities of all binding sites that lie within the region relative to TSS on the alignments that were used for creating the randomized set. We observe much smaller numbers of binding sites on the randomized promoters, e.g. only about of the motifs on the randomized alignments have more predicted sites than the motif with the least predicted sites on the true alignments. (TIFF) [file pone.0024279.s002.tiff]

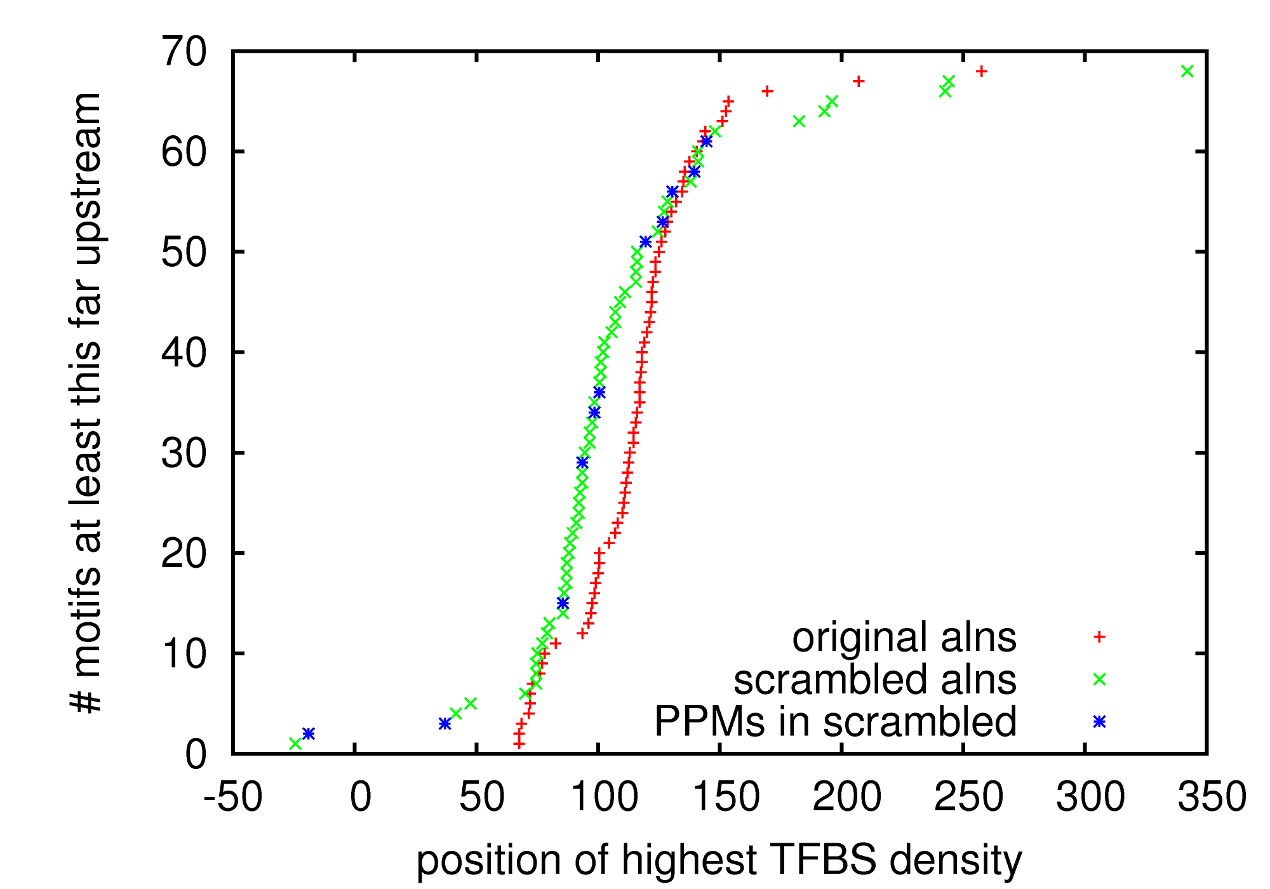

Supplement: Figure S3 — Cumulative distribution of the position of highest TFBS density across motifs for binding site predictions done on the true (red) and randomized (green) alignments. Each symbol corresponds to one motif. The blue symbols indicate the PPMs on the randomized alignments. As the figure shows, on the randomized alignments the positions of highest TFBS density for the PPMs vary greatly indicating that their preference for proximal locations in the true alignments is not a consequence of di-nucleotide composition of the promoters. (TIFF) [file pone.0024279.s003.tiff]

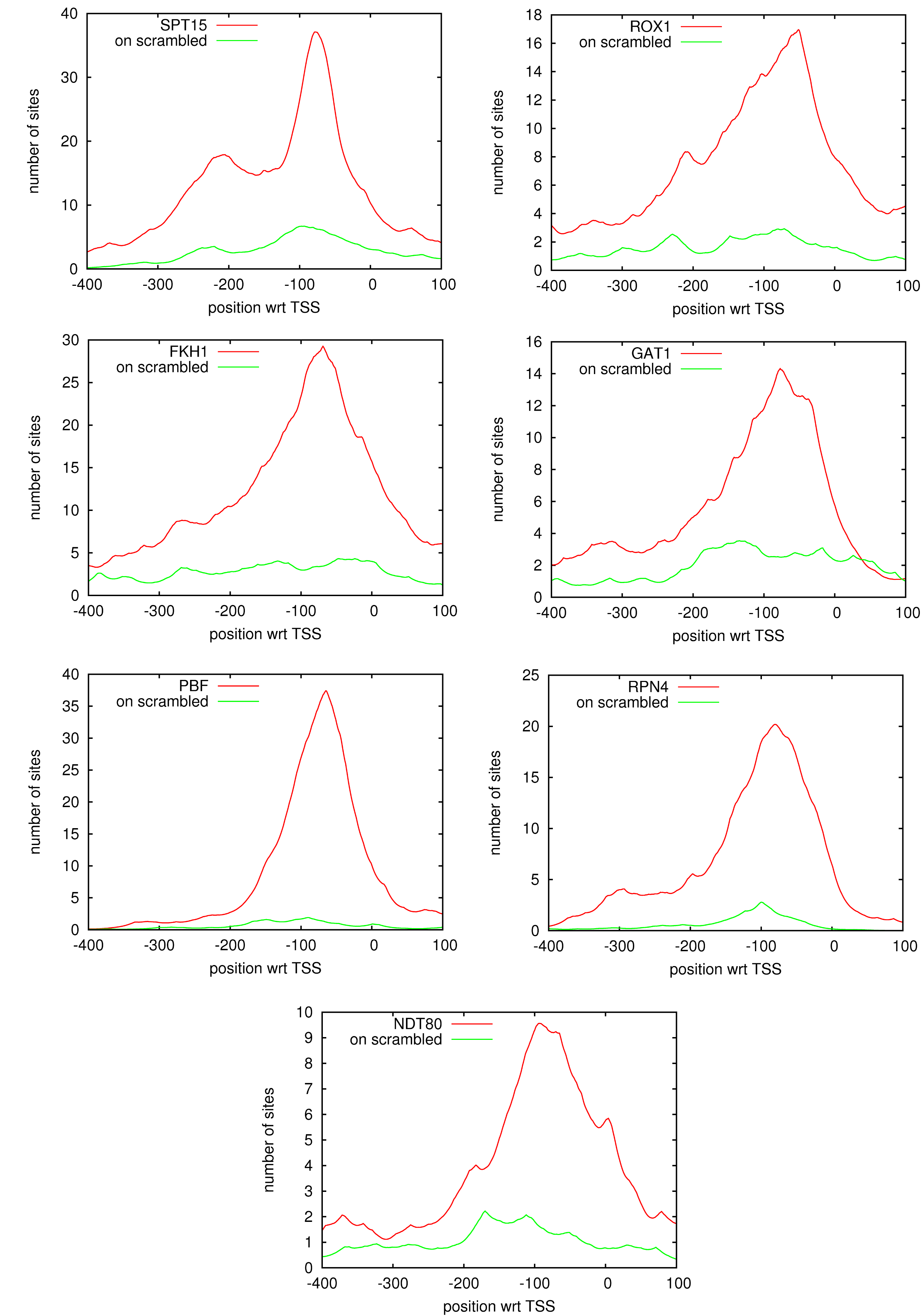

Supplement: Figure S4 — Positional distributions of TFBSs for the proximal promoter motifs on the true (red lines) and randomized (green lines) alignments. Each panel corresponds to one of the proximal promoter motifs. In each panel, the position relative to TSS is indicated along the horizontal axis and the density of TFBSs is shown along the vertical axis. The figures show that PPMs show little evidence of preferred positioning on the randomized alignments. (TIFF) [file pone.0024279.s004.tiff]

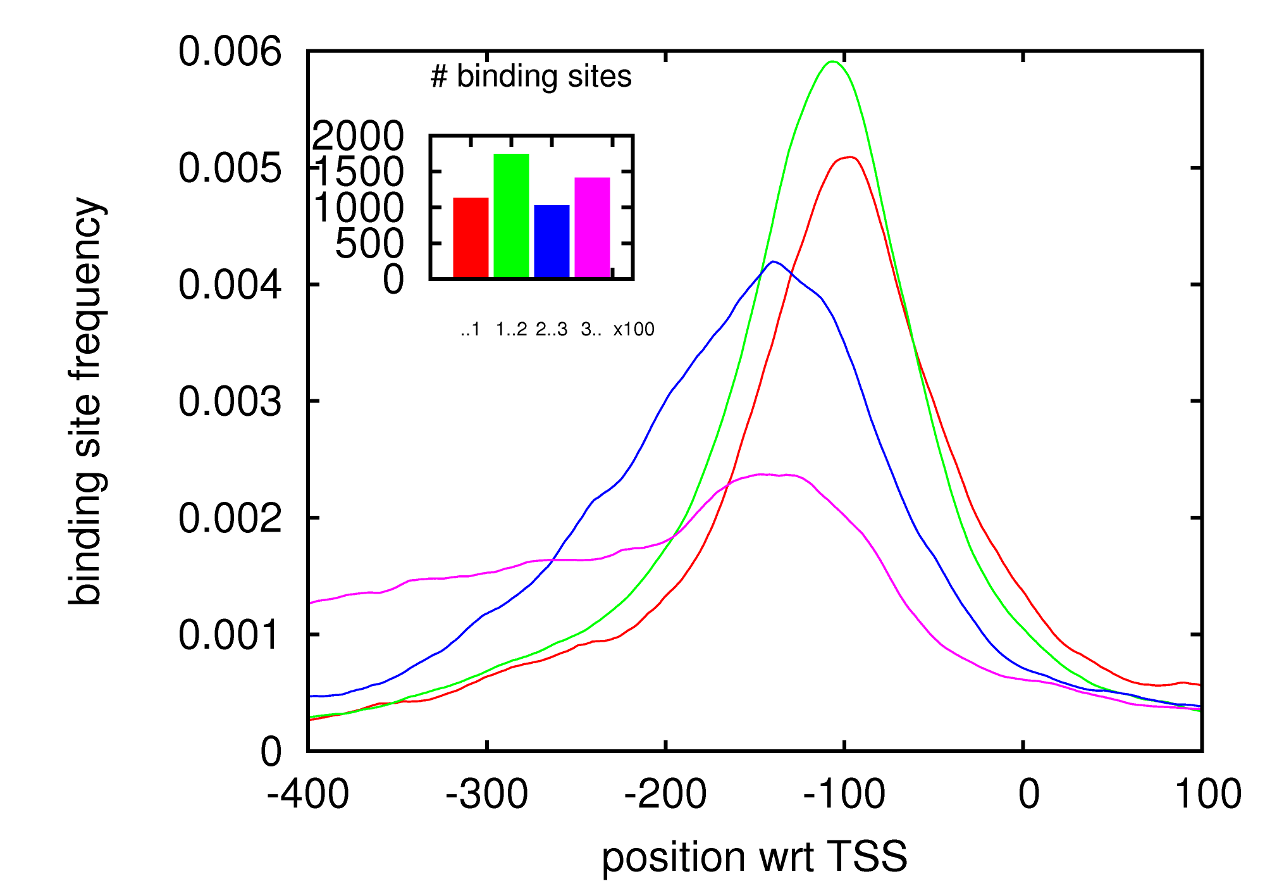

Supplement: Figure S5 — Positional distributions of all TFBSs for promoters with the region of minimal nucleosome coverage (MNO) up to 100 base pairs upstream of TSS (red), between 100 and 200 base pairs upstream (green), between 200 and 300 base pairs upstream (blue), and more than 300 base pairs upstream (purple). The inset shows the total number of binding sites in each of the promoter classes. The results demonstrate the locations of highest TFBS density match the locations of the region of minimal nucleosome occupancy. (TIFF) [file pone.0024279.s005.tiff]

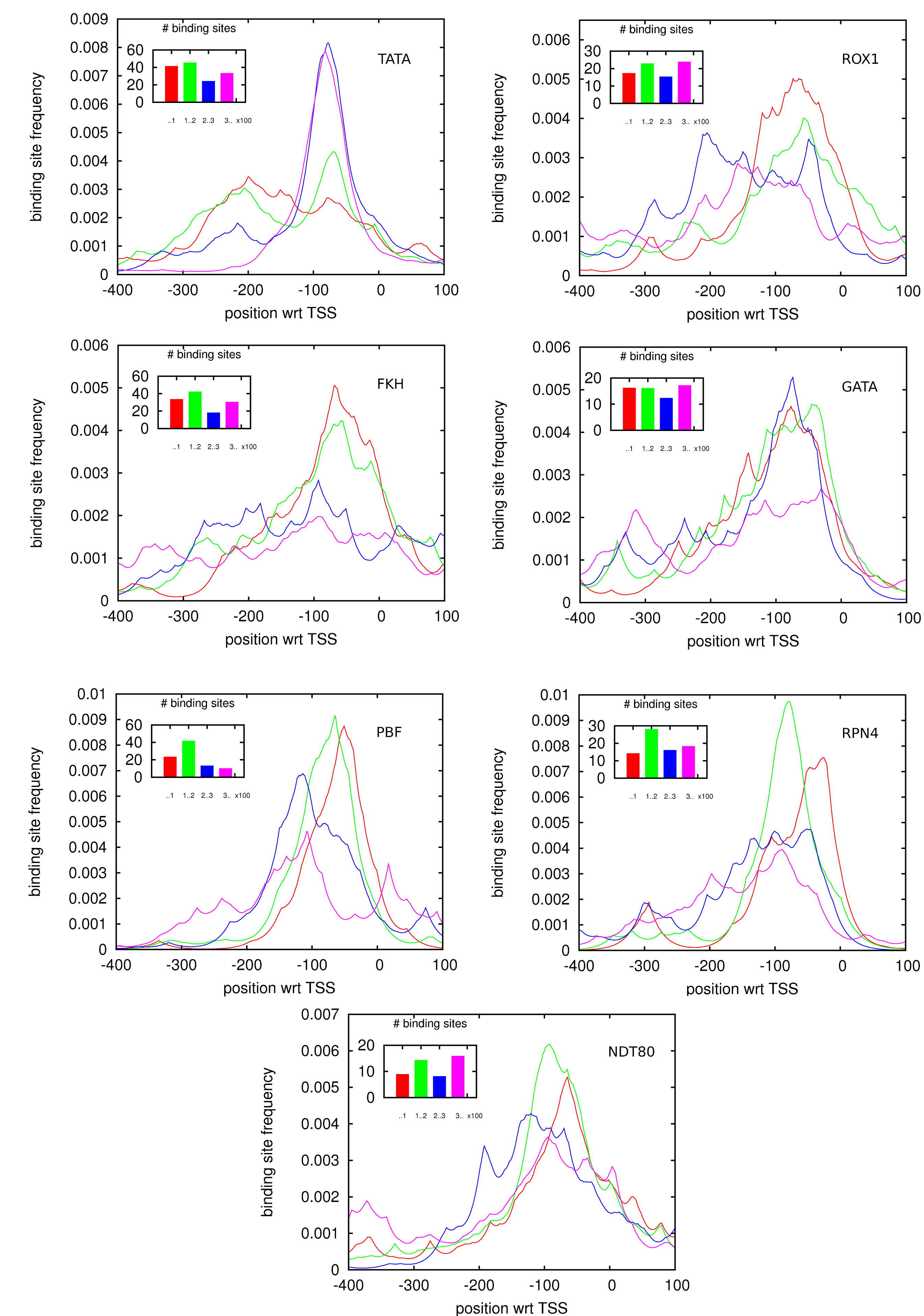

Supplement: Figure S6 — Positional distributions of the TFBSs for each proximal promoter motif, separately in promoters with the region of minimal nucleosome occupancy (MNO) up to 100 base pairs upstream of TSS (red), between 100 and 200 base pairs upstream (green), between 200 and 300 base pairs upstream (blue), and more than 300 base pairs upstream (purple). Each panel corresponds to one proximal promoter motif (first row: TATA, ROX1. Second row: FKH, GATA. Third row: PBF, RPN4. Last row: NDT80). In each panel the horizontal axis shows position relative to TSS and the vertical axis shows TFBS density. The insets show the total numbers of predicted TFBSs in each promoter class for the corresponding motif. With the exception of the TATA motif, which shows highest density of TFBSs in promoters with MNOs more than 200 base pairs upstream, all other PPMs show highest densities of TFBSs in promoters with MNOs more proximal to TSS. (TIFF) [file pone.0024279.s006.tiff]

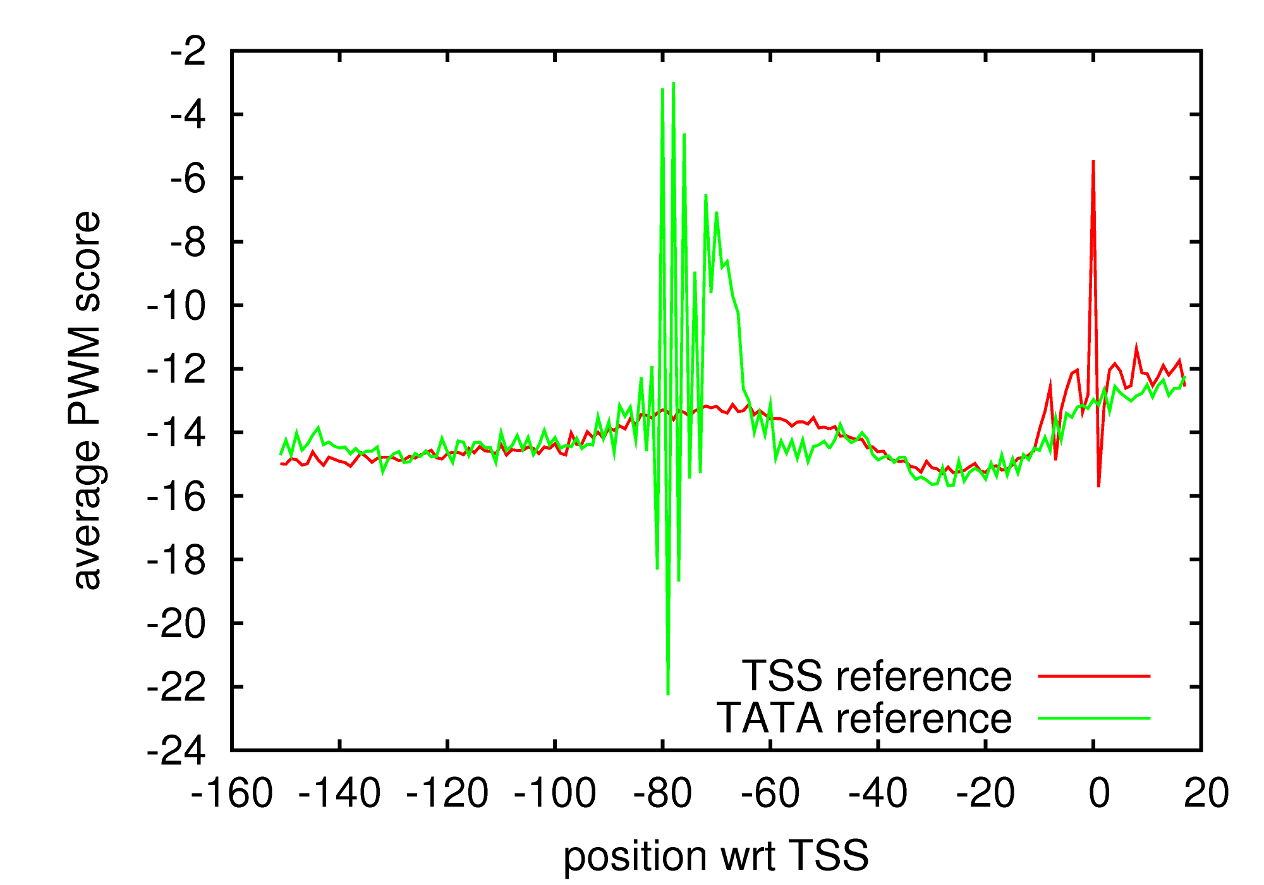

Supplement: Figure S7 — Affinity profiles for the initiator WM exclusively for TATA promoters. To construct the red profile, the TSS was taken as reference point in each promoter, whereas for the green profile the TATA box was taken as a reference point. To align the green and red profiles, we set the TATA-box reference point at the position where the highest density of TATA sites is observed ( bp relative to TSS). The results demonstrate that the second maximum of the red profile, at around bps upstream of TSS, corresponds to the affinity of the initiator motif for a region around that TATA-box. (TIFF) [file pone.0024279.s007.tiff]

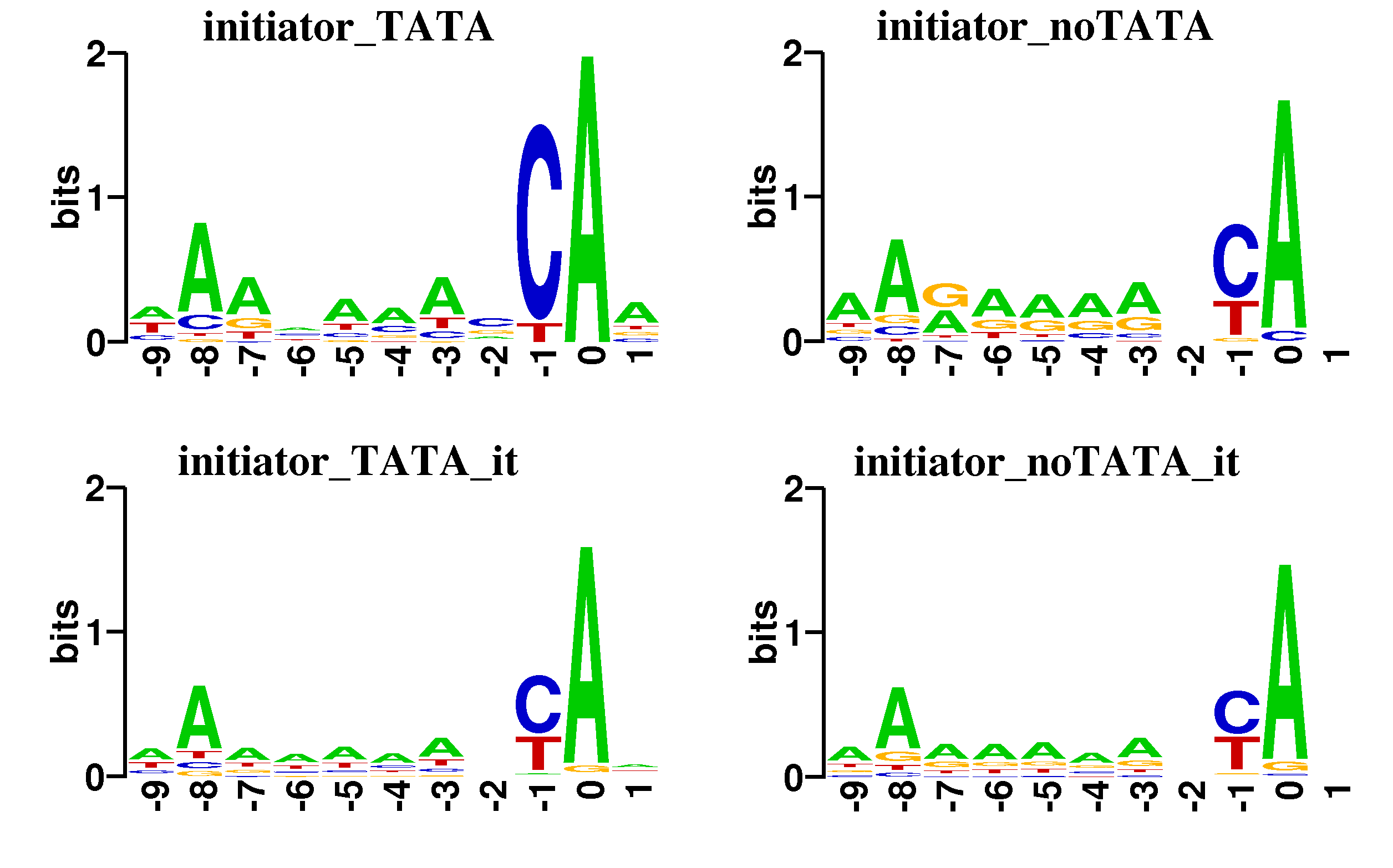

Supplement: Figure S8 — Upper panels: Seed WMs for initiator motifs in TATA and non-TATA promoters. Lower panels: Iterated WMs obtained from of the TSS sequences scoring best using the WMs in the upper panels. (TIFF) [file pone.0024279.s008.tiff]
